# Methods to identify the target population: implications for prescribing quality indicators

**DOI:** 10.1186/1472-6963-10-137

**Published:** 2010-05-26

**Authors:** Liana Martirosyan, Onyebuchi A Arah, Flora M Haaijer-Ruskamp, Jozé Braspenning, Petra Denig

**Affiliations:** 1 Department of Clinical Pharmacology, University Medical Center Groningen, University of Groningen, Groningen, the Netherlands; 2 Department of Epidemiology, University of California, Los Angeles (UCLA), School of Public Health, Los Angeles, California, USA; 3 Scientific Institute for quality of Healthcare, Radboud University Nijmegen Medical Centre, Nijmegen, the Netherlands

## Abstract

**Background:**

Information on prescribing quality is increasingly used by policy makers, insurance companies and health care providers. For reliable assessment of prescribing quality it is important to correctly identify the patients eligible for recommended treatment. Often either diagnostic codes or clinical measurements are used to identify such patients. We compared these two approaches regarding the outcome of the prescribing quality assessment and their ability to identify treated and undertreated patients.

**Methods:**

The approaches were compared using electronic health records for 3214 diabetes patients from 70 general practitioners. We selected three existing prescribing quality indicators (PQI) assessing different aspects of treatment in patients with hypertension or who were overweight. We compared population level prescribing quality scores and proportions of identified patients using definitions of hypertension or being overweight based on diagnostic codes, clinical measurements or both.

**Results:**

The prescribing quality score for prescribing any antihypertensive treatment was 93% (95% confidence interval 90-95%) using the diagnostic code-based approach, and 81% (78-83%) using the measurement-based approach. Patients receiving antihypertensive treatment had a better registration of their diagnosis compared to hypertensive patients in whom such treatment was not initiated. Scores on the other two PQI were similar for the different approaches, ranging from 64 to 66%. For all PQI, the clinical measurement -based approach identified higher proportions of both well treated and undertreated patients compared to the diagnostic code -based approach.

**Conclusions:**

The use of clinical measurements is recommended when PQI are used to identify undertreated patients. Using diagnostic codes or clinical measurement values has little impact on the outcomes of proportion-based PQI when both numerator and denominator are equally affected. In situations when a diagnosis is better registered for treated than untreated patients, as we observed for hypertension, the diagnostic code-based approach results in overestimation of provided treatment.

## Background

In the last decade, many prescribing quality indicators (PQI) have been developed to measure whether the right drugs are prescribed to the right patients [1]. They are being used for quality improvement initiatives, and to identify and reward providers who meet predefined standards of quality. For assessing prescribing quality, it is important to correctly identify the target population, *i.e*. patients with a specific condition who should receive a specific treatment. The validity of such identification depends not only on source of data but also on the type of information used to define a condition [2].

It has been recognized that the data source used can influence the outcome of the quality assessment. Administrative data, which are created mainly for billing purposes, often do not provide sufficient detail for reliable quality assessment [3-5]. Medical records provide a good alternative since they contain more detailed information although it can be difficult to extract all relevant information from this data source [6]. Aside from the data source, the operational definition of a condition may influence the outcome of the quality assessment. To identify the target population, *i.e*. patients in need of a specific treatment, either diagnostic codes or clinical measurements indicative of a disease or condition can be used. For example, to assess the quality of treatment in patients with hypertension, one can calculate the percentage of patients with the diagnosis of hypertension prescribed the recommended treatment [7-10], or the percentage of patients with elevated blood pressure levels being prescribed the recommended treatment [11-14].

These different approaches to define the target population give rise to several possible problems. Using information from recorded diagnoses can introduce bias due to incomplete registration when some patients with a condition do not have a corresponding diagnostic code registered in the data or due to incorrectly registered diagnostic codes[15,16]. Missing eligible patients is especially problematic for internal quality assessment, when health care providers use PQI as screening tools to identify patients who may benefit from the improved treatment. Using clinical measurements, on the other hand, may lead to missing patients with well-controlled disease states. In both cases, incorrect estimates of prescribing quality can occur when the accuracy of identification is not equal for treated and untreated patients. If this bias varies between providers, it can introduce misclassification on provider level and mislead pay-for-performance programs when better score on quality indicators is linked to financial incentives.

Little is known about the impact of the chosen approach to define the target population on the assessment of prescribing quality. The objective of our study was to compare the approach based on diagnostic codes registered in electronic health records (EHR) to the approach based on clinical measurements registered in EHR addressing the following questions:

1. Does the chosen approach affect prescribing quality scores?

2. What is the ability of the two approaches to identify well treated and undertreated patients?

For this study, existing PQI were selected focusing on glucose lowering and antihypertensive treatment in patients with type 2 diabetes mellitus. This is a field where both internal and external quality assessment are becoming priority for health care systems, and knowledge about the impact of the chosen approach to define the target population is important for accurate and meaningful quality measurement.

## Methods

### Study setting and sample

In The Netherlands, patients are registered with a single general practitioner (GP) who has a gatekeeper role in coordinating their medical care. Almost all GPs used electronic health records. For our study, we used data from all T2DM patients registered with 70 GPs working in 37 practices in the North of the Netherlands. These GPs participate in the GIANTT project that collects routinely documented data, such as demographics, prescribed medication, diagnoses and clinical measurements, from the EHR of the patients. An approval to use the data for this study was obtained from the Steering Committee of the GIANTT project was obtained on April 7, 2006.

Patients with T2DM were identified through screening of the electronic medical records of the GPs using text terms for diabetes (including diab*, dm, type 2, type II), diagnostic codes for diabetes (ICPC-code T90.x) [17], record flags for diabetes, and diabetes medication (ATC-code A10) [18]. All identified patients were classified by a research assistant and verified by their GP as having type 2 diabetes mellitus using the WHO classification of diabetes [19]. In general, T2DM patients visit their GP every three months, and routine blood pressure measurements are usually conducted during these visits.

### Data collection

An automatic data extraction method was used which was described previously, and is very sensitive (97-100%) in detecting relevant clinical measurement information, e.g. blood pressure and body mass index (BMI) values, irrespective of registration method or information system used by the GP [20]. The method relies on text recognition to ensure retrieval of information from 'free text' segments of the records in addition to data collection from structured tables, comparable to a manual chart review. Diagnoses are collected from the problem lists in the EHR where the GPs document medical problems pertaining to the patient using either the International Classification for Primary Care (ICPC) [17] coding or text lines, which were manually recoded into the corresponding ICPC codes by two researchers verified by an experienced GP. All participating GPs prescribed electronically, which means that the dataset included full information regarding prescribed medication.

### Prescribing quality indicators (PQI)

We included PQI that have been developed for assessing prescribing quality in T2DM patients. These PQI were derived from evidence-based diabetes guidelines, and previously tested in expert panels [9,10,21,22]. For this study, we selected two PQI focusing on the treatment of patients with hypertension and one PQI focusing on glucose management in obese or overweight patients. Both hypertension and overweight can be defined using diagnostic codes or clinical measurement values, and the required information is commonly available in the EHR (table 1). These three PQI represent different aspects of prescribing in different subgroups of T2DM patients. For the first indicator (PQI-1), the clinical measurement is directly influenced by the recommended treatment which may result in missing patients with a well-controlled disease state when using clinical measurements. This is partly the case for PQI-2, although the recommended β-blocker may not be the main treatment prescribed for lowering the blood pressure. For PQI-3, there is no direct effect of the recommended treatment on the control of the condition.

**Table 1:** Definitions of the PQI according to the diagnostic code-based approach, the clinical measurement-based approach, and the reference method

	Diagnostic code-based	Clinical measurement-based	Reference (hybrid) method
PQI-1	Denominator: T2DM patients with diagnostic codes for hypertension	Denominator: T2DM patients with SBP≥140 mmHg	Denominator: T2DM patients with diagnostic codes for of hypertension OR SBP≥140 mmHg
	Numerator: Denominator AND prescription of any antihypertensive medication	Numerator: Denominator AND prescription of any antihypertensive medication	Numerator: Denominator AND prescription of any antihypertensive medication

PQI-2	Denominator: T2DM patients with diagnostic codes for hypertension and history of IHD or MI	Denominator: T2DM patients with SBP≥140 mmHg AND history of IHD or MI	Denominator: T2DM patients with diagnostic codes for hypertension OR SBP≥140 mmHg AND history of IHD or MI
	Numerator: denominator AND prescription of beta blocker	Numerator: Denominator AND prescription of beta blocker	Numerator: Denominator AND prescription of beta blocker

PQI-3	Denominator: T2DM patients with diagnostic codes for overweight OR obesity	Denominator: T2DM patients with BMI ≥25	Denominator: T2DM patients with diagnostic codes for overweight OR obesity OR BMI≥25
	Numerator: Denominator AND prescription of metformin	Numerator: denominator AND prescription of metformin	Numerator: denominator AND prescription of metformin

T2DM: Type 2 Diabetes Mellitus, IHD: Ischaemic Heart Disease, MI: myocardial infarction, SBP: Systolic Blood Pressure, BMI: Body Mass Index (weight in kilograms divided by height in meters-squared)

### Data analysis

All the analyses were conducted using data from the EHR. The PQI were calculated using prescribing information from the second half of 2004. All preceding diagnosis information regarding hypertension (ICPC-codes K85, K86 and K87) and overweight or obesity (ICPC-codes T82 and T83) was used for the diagnostic code-based approach. For the clinical measurement-based approach, an average systolic blood pressure (SBP) of ≥140 mmHg during the first half of 2004 was used to define hypertensive patients, and the most recent BMI value in 2004 being ≥25 was used to define overweight patients. We used an average SBP ≥140 mmHg as a cut off value to identify patients with hypertension following the recommendations for treatment of T2DM patients with hypertension described in the Dutch Hypertension Guidelines for General Practitioners [23].

To check whether the inclusion of patients with an 'average' of only one elevated SBP value in the study period might be unjustified, we assessed how many of such patients had no preceding or next SBP values ≥140 mm/Hg. This was the case for only 2% of the patients with elevated average SBP levels in the first half of 2004.

To select T2DM patients with history of ischemic heart disease or myocardial infarction (PQI-2) we have used ICPC codes K74, K76, and K75. All analyses were conducted using SPSS version 16.0 (SPSS, Inc., Chicago, Illinois).

To answer our first question, we calculated the PQI scores with 95% confidence intervals using only diagnostic codes or only clinical measurement values. The unit of analysis for calculation of the PQI scores was an individual patient, therefore the prescribing quality scores discussed in this paper are population level scores. We used mixed model analysis to adjust the scores of PQI and their 95% confidence intervals for correlation within GP practices. For our second question, we calculated the ability of each approach to identify 'well treated' patients (patients receiving the treatment as recommended), and 'undertreated' patients (patients in need of treatment but not receiving the recommended treatment). This was expressed as the proportion of 'well treated' (respectively 'undertreated') patients identified with either approach from the total number of 'well treated' (respectively 'undertreated') patients identified with the reference method, where we combined diagnostic codes with clinical measurement values (box1).

Finally, we repeated the analyses in a subset of patients that had at least one registered blood pressure or BMI value during the study period to assess the impact of incomplete registration of clinical measurements on the comparison of the two approaches.

## Results

The dataset included 3214 T2DM primary care patients with an average age of 67 years and diabetes duration of 6 years; 55% were women (Table 2). Of the patients, 32% had a registered diagnosis of hypertension, and 7% had a diagnosis of overweight. Blood pressure measurements were available for 80% of the patients, and BMI measurements for 66% of patients. Among patients with registered measurements, 55% had an average systolic blood pressure ≥140 mmHg, and 55% had a BMI ≥25.

**Table 2:** Characteristics of the study population (N = 3214)

Characteristic	Value
Age, mean in years (SD)	67.1 (12.6)

Women, n (%)	1767 (55.0)

Duration of diabetes, mean in years (SD)	6.0 (5.6)

	

Registered diagnosis of hypertension, n (%)	984 (31.0)

Registered diagnosis of overweight or obesity, n (%)	213 (6.6)

Registered diagnosis of IHD or MI, n (%)	367 (11)

Registered systolic blood pressure, n (%)	2566 (79.8)

Registered Body Mass Index, n (%)	2106 (65.5)

	

Systolic blood pressure ≥140 mmHg, n (%)	1749 (54.4)

Body Mass Index ≥25, n (%)	1767 (55.0)

IHD: Ischaemic heart disease

MI: Myocardial infarction

Concurrence between registration of diagnostic codes and the corresponding clinical measurements was low. Among patients with an elevated systolic blood pressure, 62% (1086) did not have a registered diagnostic code for hypertension. In case of overweight, 92% (1624) of patients with BMI ≥25 did not have a registration of a corresponding diagnostic code (table 3).

**Table 3:** Eligibility agreement between registration of diagnoses and clinical measurements

	Diagnosis	Diagnosis	Total
	Yes	No	
SBP ≥140	663	1086	1749

SBP < 140	190	627	817

No SPB	131	517	648

Total	984	2230	3214

BMI ≥25	143	1624	1767

BMI < 25	5	334	339

No BMI	65	1043	1108

Total	213	3001	3214

SBP: Systolic Blood Pressure

BMI: Body Mass Index (weight in kilograms divided by height in meters-squared)

### Scores of PQI

The choice of approach affected the outcome of only PQI-1 focusing in prescription of any antihypertensive treatment. For this PQI the diagnostic code-based approach resulted in 12% higher prescribing quality score than measurement-based approach. For the remaining two indicators, the prescribing scores observed with different approaches were nearly identical (table 4).

**Table 4:** Scores of prescribing quality indicators identified with different approaches

Prescribing quality indicators (PQI)	Outcome of the PQI, %, (95% CI) numerator/denominator	
	Diagnostic code-based	Measurement-based
**PQI-1 **Prescription of any antihypertensive medication in hypertensive T2DM patients	93(90-95) 1412/1749	81 (78-83) 905/984

**PQI-2 **Prescription of beta blocker in hypertensive T2DM patients with a history of IHD or MI	65(57-72) 100/155	64 (56-72) 125/194

**PQI-3 **Prescription of metformin in overweight T2DM patients	65(59-72) 39/213	66(62-69) 1154/1767

T2DM: Type 2 Diabetes mellitus

IHD: Ischaemic Heart Disease

MI: Myocardial Infarction

### Ability of identifying well treated and undertreated patients

The use of either diagnostic codes or clinical measurements to identify well treated or undertreated patients resulted in absolute differences in proportions of identified patients ranging from 15% to 84% (table 5). In all cases, the measurement-based approach identified more well treated and undertreated patients than the diagnostic code-based approach. For well treated patients, the proportion identified raised from 54% (diagnostic code-based) to 84% (measurement-based) for antihypertensive treatment in general (PQI-1), from 63% to 79% for beta blocker treatment after ischemic heart diseases (PQI-2), and from 12% to 97% for metformin treatment in overweight patients (PQI-3). Similarly, the proportion of undertreated patients identified increased from 21% (diagnostic code-based) to 88% (measurement-based) for PQI-1, from 60% to 75% for PQI-2, and from 11% to 95% for PQI-3 when clinical measurements were used (table 5).

**Table 5:** Identification of well treated and undertreated patients using the diagnostic code-based and clinical measurement-based approach

	Well treated patients		Undertreated patients		
	
Type of approach	proportion detected*, %	N	Proportion detected**, %	N	N of eligible patients per PQI
**PQI-1 **Prescription of any antihypertensive medication in hypertensive T2DM patients					2070

Reference method	-	1687	-	383	
Diagnostic code-based, hypertension	54 (905/1687)	905	21 (79/383)	79	
Measurement-based, SBP ≥140	84 (1412/1687)	1412	88 (337/383)	337	

**PQI-2 **Prescription of beta blocker in hypertensive T2DM patients with a history of IHD or MI					251

Reference method	-	159	-	92	
Diagnostic code-based, hypertension	63 (100/159)	100	60 (55/92)	55	
Measurement-based, SBP ≥140	79 (125/159)	125	75 (69/92)	69	

**PQI-3 **Prescription of metformin in overweight T2DM patients					1837

Reference	-	1193	-	644	
Diagnostic code-based, overweight or obesity	12 (139/1193)	139	11 (74/644)	74	
Measurement-based, BMI ≥25	97 (1154/1193)	1154	95 (613/644)	613	

T2DM: Type 2 Diabetes mellitus

SBP: Systolic Blood Pressure

BMI: Body Mass Index (weight in kilograms divided by height in meters-squared)

*Number of treated patients detected through the tested approach divided by the number of treated patients according to the reference method

**Number of untreated patients detected through the tested approach divided by the number of untreated patients according to the reference method

Using the diagnostic code-based approach, a clear difference was observed in its ability to identify well treated versus undertreated patients for antihypertensive treatment in general (PQI-1). This approach identified 54% of the well treated but only 21% of the undertreated patients, indicating that the registration of a hypertension diagnosis in the EHR is more likely when drug treatment is initiated than when drug treatment is not (yet) initiated. Such bias was not observed for the other two PQI (table 5).

### Subset analysis

We repeated the analyses in subsets of patients that had at least one recorded blood pressure measurement for PQI-1 (1939 of the 2070 hypertensive patients) and PQI-2 (227 of the 251 hypertensive patients with IHD or MI), and at least one BMI value for PQI-3 (1772 of the 1837 overweight patients). The PQI scores for the subset were quite similar to scores observed for the whole study population. According to the reference method, the prescribing quality scores calculated for the subsets changed from 81% to 82% for PQI-1, from 63% to 64% for PQI-2, and remained 65% for PQI-3. For the diagnostic code-based approach, observed changes were 92% to 94% (PQI-1), 65% to 66% (PQI-2), 65% to 70% (PQI-3). As could be expected, the proportion of identified well treated and undertreated patients with the measurement-based approach increased for this subset of patients, and approached the reference method with proportions of 89%, 86%, 100% for well treated and 95%, 85%, 100% for undertreated patients.

## Discussion

Our study showed that prescribing quality scores do not necessarily change when using different approaches to define the number of patients eligible for treatment. However, when diagnosis is registered better for treated than for untreated patients, as was the case for hypertension, the diagnostic code-based approach resulted in overestimating the prescribing quality (93 versus 81%). In addition, it became clear that incomplete registration of diagnostic codes is a big problem for conditions such as hypertension and overweight, leading to the identification of low proportions of patients in need of treatment (11-60%) when using a diagnostic code-based approach.

In general, PQI are proportion-based measures which can be quite robust to changes in the numerator, as any change in the numerator causes changes in the denominator [24]. This was the case for the indicators focusing on the prescription of beta blockers and of metformin in specific patient groups (PQI-2 and PQI-3). However, for the indicator focusing on the prescription of any antihypertensive drug (PQI-1), the diagnostic code-based approach resulted in a higher score on prescribing quality compared to the clinical measurements-based and reference methods. The explanation of this finding is that the registration of the diagnostic codes for hypertension is more likely once antihypertensive treatment is prescribed, as was illustrated by the low percentage of untreated in comparison to treated hypertensive patients identified with the diagnostic code-based approach. A similar finding was observed in non-diabetic population, where treated patients also had a better registration of the diagnosis of hypertension [25].

In our study population, the clinical measurement-based approach identified higher proportion of patients who are in need of treatment compared to the diagnostic code-based approach. This is due to the fact that many patients with either high blood pressure or BMI levels did not have a registration of the corresponding diagnostic code in the EHR. Poor registration of conditions such as hypertension and especially overweight in the EHR seems to be a common problem [26-28]. It has therefore been advocated to use clinical measurements to improve documentation of such conditions [29]. Improved registration of diagnostic codes as a part of quality improvement programs may make diagnostic code-based PQI more reliable. It is important to realize, however, that the validity of registered diagnoses is influenced by many factors including the purpose of registration, skills and knowledge of the coder, insensitive coding schemes for registering specific diseases, prioritizing the coding of some conditions over others by physicians, and completeness of a disease classification system [15,30].

Clinical measurement values appear to be a better choice for prescribing quality assessment, especially for internal quality assurance, when it is crucial to correctly identify as many patients who could benefit from the improved treatment. When the clinical measurement values are influenced by the recommended treatment, as is the case for PQI-1 and PQI-2, a clinical measurement-based approach for assessing the treatment may result in missing patients with well-controlled disease states. This is particularly a problem when patient eligibility and prescribed treatment are assessed cross-sectionally [14]. When prescribing is assessed in a sequential way (i.e. after the observed clinical measurement), as was done in our study, missing well-controlled patients appeared not to affect the quality scores. In situations where there are already much higher percentages of well-controlled patients, however, a measurement-based approach can result in lower prescribing quality scores in comparison to a diagnostic code-based approach.

In our study we used cut off levels of SBP ≥140 mmHg to identify patients with hypertension as advised by Dutch hypertension guidelines. However, World Health Organization (WHO) and International Society of Hypertension (ISH) advised to use lower cut off levels of SBP to diagnose hypertension in T2DM patients [31]. Use of lower values of SBP to identify hypertensive patients may result in larger differences between the PQI scores when the different approaches are used.

We used a sensitive method for data abstraction from medical records. Registration of diagnostic codes was complemented by recoding diagnoses from text lines. Our reference method was based on a combination of available information about diagnosis and measurements documented in the EHR. Although EHR are often considered the gold standard for quality measurement, inadequate registration of both diagnoses and clinical measurements affects this reference method. Our subset analysis, however, showed that the prescribing quality scores were not affected by incomplete registration of clinical measurements. The PQI scores and proportions of identified patients may not be generalizable to other databases with different registration rates of clinical measurements or diagnostic codes but the identified problems are likely to occur in other settings. The registration rates in our dataset were similar to those described in other studies conducted in different parts of the world using EHR of both diabetic and general primary care population [25,32,33].

Finally, it has to be kept in mind that if these PQI are used for comparison of individual GPs, the number of eligible patients per PQI per GP may not be sufficient for reliable benchmarking. To address the problem of a small sample size per PQI, one could choose from several existing methods including pooling data from several health care providers or time periods or excluding indicators or health care providers with small patient numbers [21].

Although in our study setting the ICPC codes were used, we expect that the results of our study are also relevant for health care systems using the International Classification of Diseases (ICD), as this classification system also includes diagnostic codes for hypertension, overweight and obesity that could be combined or substituted with clinical measurement values.

## Conclusion

To our knowledge, this is the first study addressing the impact of using different types of information to define a condition on the assessment of prescribing quality. With the increasing use of electronic health records, which offer more complete information than administrative data, EHR have the potential to provide sensitive estimates of healthcare quality. Our study shows some drawbacks of using either diagnostic codes or clinical measurement values from the EHR for prescribing quality assessment. Although both approaches resulted in missing patients who could benefit from the recommended treatment, the use of clinical measurements is more sensitive to screen for poorly treated patients. This is important for quality improvement purposes. When there is information bias in the documentation of diagnoses in relation to the treatment status, the use of diagnostic codes alone can mislead both policy makers and health care providers about the performance scores of quality indicators. In such cases, a combination of diagnostic codes and clinical measurement information is recommended for prescribing quality assessment.

## Competing interests

The authors declare that they have no competing interests.

## Authors' contributions

LM originated the idea for this study, did the research proposal, data analysis, and prepared the manuscript. PD and HR-FM contributed to the research proposal, reviewed the analysis, and participated in the preparation of the manuscript. JB participated in the interpretation of the data and in the discussion of the paper. OAA participated in the data analyses and edited and reviewed the manuscript. All authors read and approved the final manuscript.

## Pre-publication history

The pre-publication history for this paper can be accessed here:

http://www.biomedcentral.com/1472-6963/10/137/prepub
